# Development of health behavior change for early adulthood based on situational expectancy-value theory: study protocol for the Haian Flower Drums Project

**DOI:** 10.3389/fpubh.2026.1814656

**Published:** 2026-05-07

**Authors:** Jiangping Fu, Yi Yu, Chunfang Zhao, Bing Cao, Zhi Sun

**Affiliations:** 1 Nantong Institute of Technology, Nantong, Jiangsu, China; 2 Zhengde Polytechnic, Nanjing, Jiangsu, China; 3 Jiangsu Open University, Nanjing, Jiangsu, China

**Keywords:** early adulthood, expectancy-value theory, Haian Flower Drum, health behavior change, physical activity, physical literacy

## Abstract

**Background:**

As an effective physical activity strategy and modality, Intangible Cultural Heritage Sport Program (ICHSP) may be an important direction for accessibility research for quality healthcare and quality education. It is about exploring the feasibility and sensitivity of developing the Haian Flower Drums Project (HFDP) based on situated expectancy-value theory (SEVT) as an intervention for health behavior change (HBC) in school-based physical education for university students in early adulthood.

**Methods:**

This study protocol was a two-arm (604 university students) 64-week randomized controlled trial with a 3-year follow-up of a single-arm longitudinal cohort at prognosis after the intervention ended. The SEVT-based Haian Fower Drum Project was used as an experimental intervention and to provide a structural model. The primary outcomes were the validity, feasibility, and sensitivity of the structural model, which incorporated university students' academic level (independent variable), physical literacy (dependent variable), and the Teach-Practice-Play model (physical activity, covariate). The secondary outcome is an exploratory analysis of the psycho-structural characteristics of university students during their HBC. Physical literacy data will be collected throughout the study (t0–t5) to conduct exploratory analyses of the intervention's or structural model model's effectiveness. Demographic information and academic level (physical fitness measurements) will also be collected during the t0–t4 phases, and post-intervention satisfaction (wellbeing, self-efficacy, social support, health perceptions, and stress perception) will be assessed during the t3–t4 phases for the Haian Flower Drum Project to validate its feasibility and sensitivity, as well as to conduct an exploratory analysis of the psychological structural characteristics in the HBC process among university students. Results for the primary outcome will be analyzed using linear mixed models. The results of the secondary outcomes will be analyzed using non-parametric variables (Spearman correlations).

**Discussion:**

This study protocol focuses on the feasibility of promoting mental health and the psychosocial characteristics of ICHS in the context of HBC in early adulthood.

**Anticipated results:**

The SEVT-based Haian Flower Drum Project provides a structural model that may serve as strong evidence for physical activity in early adulthood.

**Clinical trial registration:**

https://www.chictr.org.cn/showproj.html?proj=278225, identifier: ChiCTR2500110409.

## 1 Introduction

In public health, health behavior change (HBC) has emerged as an effective prevention strategy for promoting effective changes in physical activity, particularly in the context of chronic diseases (1). As an effective prevention strategy, health behavior change is even more important for accessibility research for quality healthcare and quality education (2). Health behavior change, in both longitudinal and cross-sectional age-series studies, has always been a hot topic in shaping lifelong health (3). A 2024 prospective study of delinquent behavior and obesity in adolescents concluded that health behavior change is difficult (4). Previous studies have established that physical activity in early adulthood plays a critical role in shaping lifelong health outcomes. Still, its short-, medium-, and long-term benefits for health behavior change require sustained attention.

The process of health behavior change has presented physical literacy as a beneficial concept to counter sedentary behavior and reduced physical activity (5). A study of physical activity in early adulthood (college students) suggests that being physically active is an important mediator of health behavior change (physical literacy vs. amount of exercise in physical activity) (6). General practitioners help patients' health behavior change and enhance their wellbeing through green exercise prescriptions, such as physical activity in the natural environment (7). Completed and ongoing research on the topic of shaping active lives may suggest that physical literacy, physical activity, health behavior, and the environment form a situational grouping of health behavior change. This situational grouping, an important factor in the short-, medium-, and long-term benefits of health behavior change, should benefit public health by promoting lifelong health and wellbeing.

Scholars conduct a variety of health behavior change interventions and physical activity modeling to shape active living. 2025 A recent pilot study of the effects of a national sport (Tai Chi stick) based health behavior change program on physical activity showed that an 11-week intervention program was effective in improving physical activity (8). In 2025, a study explained how the Intangible Cultural Heritage Sports Program (ICHSP), operating as a sustainable development model, provides a series of risk indicators for cultural vitality, offering participants a reference for the benefits and influencing factors of health behavior changes (9). A physical activity intervention in a school soccer sports program demonstrated the importance of health behavior change (sustained participation) in early adulthood (10). The cultural characteristics of sports programs that focus on health behavior change (physical activity to health behavior) in early adulthood may be a common feature of physical activity that shapes active living. At the same time, health behavior change may also be a reason, as well as a gap, why physical activity remains a research hotspot in academia. ICHSP may be an effective method or means of health behavior change and should prove beneficial to public health throughout life.

At the theoretical level, the situated expectancy-value theory (SEVT) posits that health behavior change in early adulthood is associated with expectancy and value, thereby motivating behavioral stability and addressing the difficulty of health behavior change (11). SEVT addresses the environmental grouping of health behavior change by focusing on capabilities (physical activity/health behavior), perceptions of value, and cost beliefs in early adult scenarios (12). At the policy level, the Chinese government has proposed the 2030 Physical Literacy Indicators as a guide to expectations for health behavior change in early adulthood (13). At the level of education and teaching, the Chinese government has proposed the 2030 Strategy for a Stronger Education, which gives the “Teach-Practice-Play” physical activity model (TPPPAM) for physical education as a guideline for the operational value of health behavior change in early adulthood (13, 14). On a practical level, ICHSP, with its characteristic physical culture (clothing, equipment, and movement combinations) activity, has become an effective way to address the situational grouping of health behavior change as a physical activity program in early adulthood (15–19). Based on complexity theory (20), the behavioral changes in health in early adulthood toward a trajectory of physical activity still have gaps and critical issues. Based on the information in this paragraph, it may be confirmed that a physical activity/ICHSP based on the SEVT design may be effective for perceived physical literacy and health behavior change in early adulthood.

Based on the strengths of previous studies and the gaps in health behavior change in early adulthood, this study protocol presents its own research objectives/anticipated results. To put it more clearly, this study takes the Haian Flower Drum Project (HFDP) as an example (21). As a type of physical activity/ICHSP within the SEVT framework, its cultural activity possesses the expected attributes for health behavior change in early adulthood, while its physical activity possesses the value attributes for such change. Meanwhile, TPPPAM enhances confidence in success during early adulthood (through step-by-step goal setting) and reinforces a sense of learning value (by linking it to future careers). In other words, university students in early adulthood—the target audience—exhibit high motivation to participate in the Haian Flower Drum Project during the initial stages of health behavior change. The question is whether, under the influence of TPPPAM, this health behavior change can evolve into a psychological characteristic of stable motivation. Based on this, the objectives of this study protocol include 1 key objective and 5 secondary objectives.

### 1.1 Key objective

The key objective of this study protocol was to explore the feasibility and sensitivity of an ICHSP [SEVT-based Haian Flower Drum Project (HFDP), (21)] as an intervention for health behavior change in school physical education for university students in early adulthood.

### 1.2 Secondary objective 1

HFDP may be an effective approach for a cross-sectional study of health behavior change to observe the psycho-structural characteristics of health behavior change in early adulthood.

### 1.3 Secondary objective 2

The impact of HFDP on perceived physical literacy among university students may serve as a means to assess the feasibility of health behavior change and is related to physical activity and academic level.

### 1.4 Secondary objectives 3

To observe the psychological structural characteristics of short-term benefits of HBC in early adulthood under an intervention based on the SEVT-designed HFDP, and to determine whether physical literacy may be related to self-efficacy and perceived stress.

### 1.5 Secondary objective 4

To observe the psycho-structural characteristics of the mid-term benefits of HBC in early adulthood under an intervention based on the HFDP designed by SEVT, and that physical literacy may be related to social support, health perceptions, and wellbeing.

### 1.6 Secondary objective 5

To observe the characteristics of psycho-structural changes in HBC in early adulthood under the intervention of HFDP based on SEVT design.

## 2 Methods and analysis

**Figure 1:**
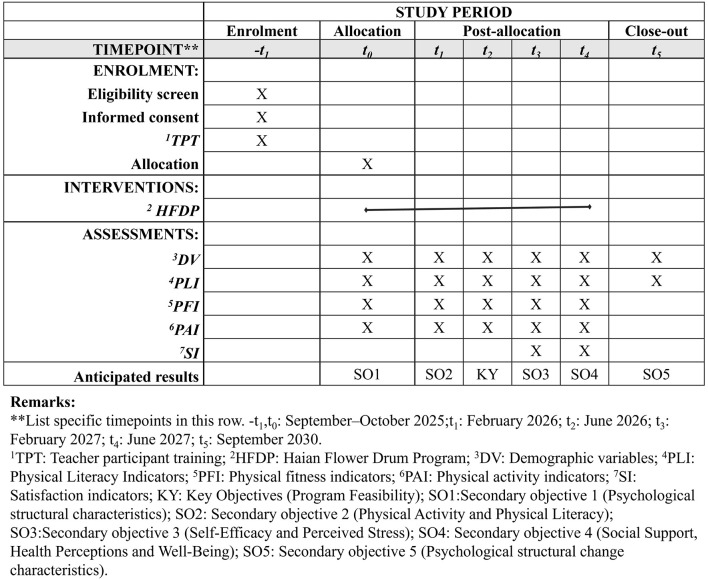
Intervention flowchart for Haian Flower Drum Project.

This study protocol was strictly implemented and followed the SPIRIT 2013 (22) and CONSORT 2025 guidelines (23) to develop the (Figure 1) flowchart.

### 2.1 Study protocol design

This study protocol was a two-arm, 64-week, randomized controlled trial with a 3-year follow-up of a single-arm longitudinal cohort to assess prognosis after the intervention ended.

University students aged 18 and over will be recruited for the study and assigned to either the experimental group or the control group. Participants will provide informed consent and complete pre-intervention assessments; thereafter, in addition to participating in regular physical education classes, the experimental group will take part in the Haian Flower Drum Project, while the control group will not.

**Figure 2:**
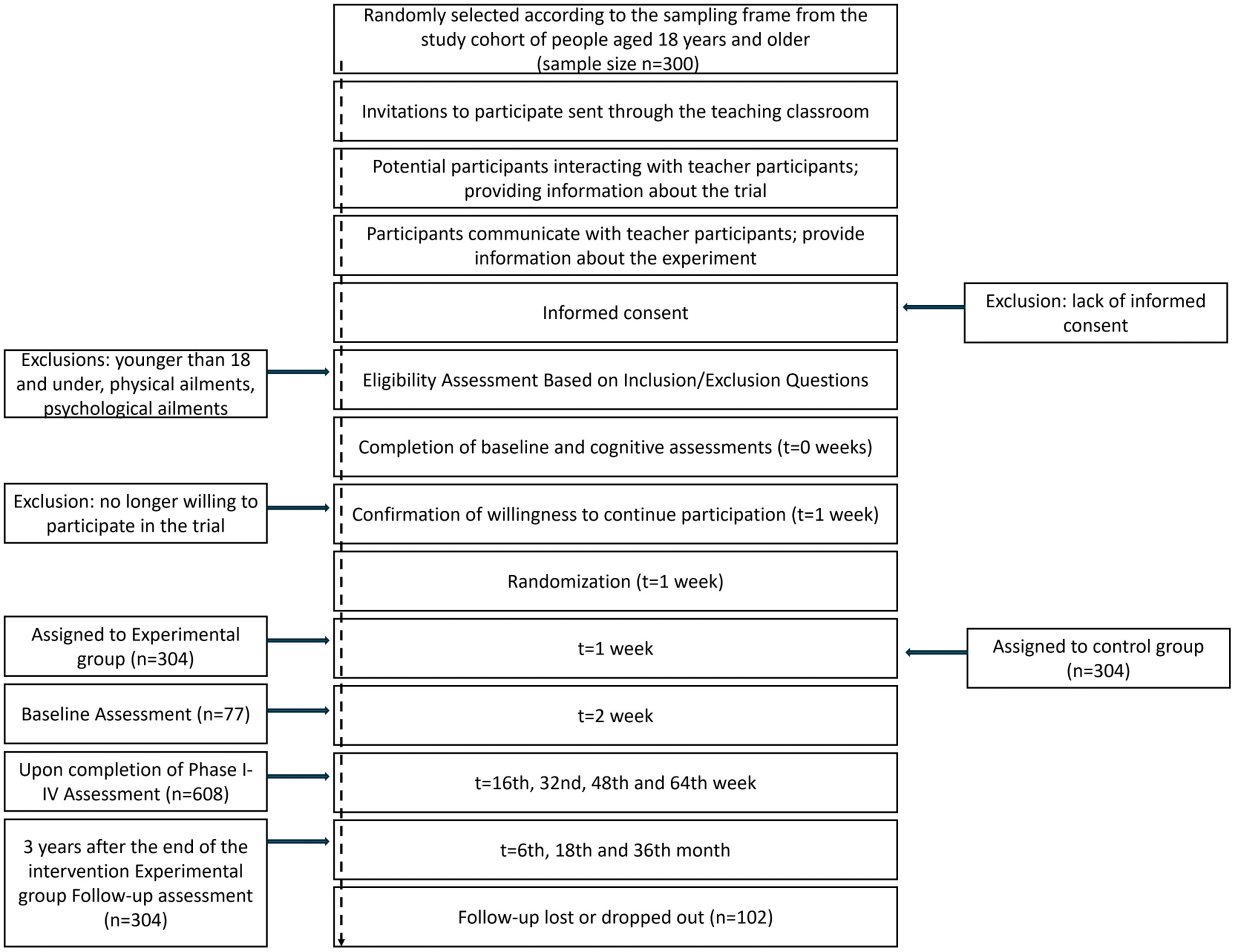
Schedule for registration, intervention, and evaluation of the Haian Flower Drum Project.

The primary outcome of this study protocol was the validity, feasibility, and sensitivity of the HFDP (Key objective), and the secondary outcome was the observation of the psycho-structural characteristics of the health behavior change process in university students (Secondary objectives 1–5; in the experiment, short term, medium term, and change). See Figure 2.

In this study protocol, the health behavior changes structural model of HFDP based on SEVT was set up, where the independent variable was the academic level of college students participating in the Haian Flower Drums (data from the physical fitness test), the dependent variable was physical literacy. The covariates were TPPPAM (time, speed, distance, and energy consumed in physical activity).

Physical literacy data will be collected throughout the study (t0–t5) to conduct exploratory analyses of the intervention's or the structural model's effectiveness. Demographic information and academic performance (physical fitness measurements) will also be collected during the t0–t4 phases, and post-intervention satisfaction (wellbeing, self-efficacy, social support, health perceptions, and stress perception) will be assessed during the t3–t4 phases for the Haian Flower Drum Project to validate its feasibility and sensitivity, as well as to conduct an exploratory analysis of the psycho-structural characteristics in the health behavior change process among university students.

It should be noted that, following the intervention completion, a 3-year single-arm longitudinal cohort follow-up was conducted exclusively in the experimental group. Physical literacy data were collected at 6th, 18th, and 36th months to explore the time-series psycho-structural characteristics underlying university students' health behavior changes through exploratory analysis.

#### 2.1.1 Adverse events/reactions

Two adverse events and two adverse reactions may have occurred in this study protocol.

Adverse Event 1, Physical Activity Hyperlocal: Any adverse medical event (symptom/sign/laboratory abnormality) occurring in a subject during the treatment period due to excessive self-imposed exercise load [threshold/heart rate range for exercise load = (225/220 – age) × (0.6 – 0.8)] during the course of the physical activity, whether or not related to the intervention.

Adverse Event 2, Physical Activity Safety: Any adverse medical event (signs/symptoms/laboratory abnormalities) occurring in the subject during the treatment period due to the exercise environment, venue facilities during physical activity, whether or not related to the intervention.

Adverse Reactions 1, Low Questionnaire Validity: Adverse events for which the validity of the questionnaire (α, less than 0.7) was assessed to confirm a causal relationship with the psychological intervention.

Adverse Reaction 2, Physical Activity Adherence Instability: Adverse events of physical activity adherence (attendance, less than 0.33) that were assessed to confirm a causal relationship with the psychological intervention.

### 2.2 Selection/treatment of subjects

#### 2.2.1 Background setting

This study protocol will be conducted with three universities in Jiangsu Province, China, and university students will be recruited as participants.

#### 2.2.2 Inclusion/exclusion criteria

Inclusion criteria included the following five: (1) 18 years and older; (2) university students; (3) consent to the program intervention and signing of an informed acknowledgment; (4) a history of physical activity; and (5) no psychological disorders, disabilities, or periods of rehabilitative care.

Exclusion criteria included the following five: (1) younger than 18 years of age or younger; (2) did not consent to the program intervention or did not sign an acknowledgment form; (3) had a psychological disorder; (4) had a physical disorder that prevented them from participating in the physical fitness test; and (5) withdrew from the program/suspended/no longer agreed to the participant.

#### 2.2.3 Participants recruitment

Recruitment of participants and the signing of informed consent forms were conducted in September–October 2025, following the agreement of all participating units (Department of Physical Education). The recruitment took place at the school's Department of Physical Education. For the recruitment method, the Department of Physical Education recommended three persons as teacher participants, and each teacher participant recommended one researcher (experimental group collector) to implement the collection. Screening was conducted by both the research director and the experimental director.

#### 2.2.4 Participant treatment

##### 2.2.4.1 Experimental group

Participants were asked to engage in activities 3 times per week during the program's intervention, and a gravity gas pedal was used to monitor and record indicators of physical activity. The first activity was a TPPPAM teaching activity, in which the teacher participant was responsible for teaching online using the Internet, and the university students learned the techniques of the Haian Flower Drum and the culture of the sport. The second event was a TPPPAM hard practice, where university students cperformed a physical activity on Haian Flower Drums. The third event is a regular TPPPAM event where university students participate in extracurricular performances or competitions.

##### 2.2.4.2 Control group

The control group consists of two sub-groups: Control group A and Control group B. Control group A: During the intervention, participants were not required to engage in activities three times a week; instead, they participated only in their regular physical education classes. Control group B: The only difference in intervention compared to the experimental group was the non-Haian Flower Drum Project. Researchers provided three specific options: general physical activity (jogging), traditional sports (Tai Chi), and Intangible Cultural Heritage Sports Program (Dao Hualan).

### 2.3 Methods of intervention

#### 2.3.1 HFDP based on SEVT

For clarity and scientific rigor, the researcher defined the name the program plan in the first step of the research proposal development: the “SEVT-based HFDP,” to achieve the research objectives and program. Based on the information in the introductory section, the researcher identified the basic logic of the HFDP: the inclusion of Haian Flower Drums as the physical activity of the ICHSP, the inclusion of TPPPAM as the health behavior of university students' participation in Haian Flower Drums, the inclusion of physical literacy as the theoretical basis for health behavior change, and the inclusion of SEVT as the theoretical basis for the overall program design.

A more intuitive and concrete expression of TPPPAM is that university students learn the movement skills of Haian Flower Drums through physical education classes, then practice frequently in interest groups, and finally participate in competitions or performances in clubs to achieve health behavior change.

Given the health behavior change program's primary outcome, the definition of physical literacy is critical. Whitehead, an advocate of physical literacy, believes it consists of two stages and six dimensions: the inner core stage (motivation, physical ability, and interaction with the environment), and the outer core stage (self-confidence, expression, and understanding of knowledge) (24). Through relevant previous studies, based on the theory of perceived behavior (24–27), it is believed that the situational grouping of physical literacy includes individual situations (motivation, physical ability, and self-confidence) as well as collective situations (interaction with the environment, expression, and understanding of knowledge).

Certainly, the present study protocol establishes the hypothesis that the Haian Flower Drum, as a specific ICHSP, can contribute participants (university students in early adulthood) perceived physical literacy, forming a stable trajectory of physical activity and health behavior change. And this health behavior change may be advantageous and helpful in overcoming internal barriers (fatigue, cost, age, and lack of time) in early adulthood (28), while forming a sense of wellbeing (29), triggering self-efficacy (30), social support perceptions (31), health perceptions (32), and stress perceptions (33).

Guided by the theory of expected utility, this study focuses on the key concept of “increasing cost beliefs” as a key intervention point. By integrating core theories from the “Culture and Personality” school of cultural psychology, which posits that culture influences behavioral patterns and emotional responses by shaping individuals' psychological structures (15–19). This study establishes the TPPPAM model for physical activity. This TPPPAM model outlines a closed-loop path: perception of the cultural significance of the Haian Flower Drum Project → enhancement of physical literacy → health behavior change → generation of psychological satisfaction. It further explores how Haian Flower Drum can overcome the challenges associated with health behavior change. Specifically, the cost belief focuses on the perceived effort required for physical fitness, aiming to eliminate adverse events or reactions associated with physical activity, including time constraints, physical fatigue, and the potential social stigma associated with learning traditional folk dance. The TPPPAM model establishes an operational psychological bridge for the cultural symbol to health behavior change. The concomitant nature of satisfaction validates the universality, replicability, and scalability of the cultural motivation and emotional response in the health behavior change facilitated by the Haian Flower Drum Project.

#### 2.3.2 Procedures of intervention

Randomized grouping: this study protocol was a two-group experiment, but for the sake of scientific and content validity. A member of the research team was identified as the sub-group researcher, specialized in the sub-grouping methodology, and was not involved in the recruitment of cases (samples), and was responsible for the strict maintenance of the allocation table. The sub-group researcher used the computer software EXCEL as a tool to generate random sequences through a simple random sampling method [using a computerized random function that generates random sequences and matches them to the sample identities (IDs)]. The specific randomization process involves the researcher using a random sequence to assign all participants to an experimental group and a control group in a 1:1 ratio; the control group is then further divided into two sub-groups, A and B, also in a 1:1 ratio.

Blinding: (1) Blinding method: This study protocol was of a triple-blind type. (2) Quality control: first, regular verification of intervention records was adopted to monitor accidental blinding for blinding maintenance; second, emergency blinding rights were reserved for the Principal Investigator (if participants appeared to drop out to continue their participation). (3) Blinding process: blinding of participants: use of a numbering system instead of group labels (random coding), harmonization of the appearance of the intervention materials (uniformity in the interface of the registration system with the same interface and the use of the questionnaire star tool). In addition, participants in Control group B were provided with intervention recommendations similar to the TPPPAM program for the Haian Flower Drum Project, including general physical activity (jogging), ethnic sports (Tai Chi), and Intangible Cultural Heritage Sports Program (Dao Hualan); participants in Control group A were not required to engage in TPPPAM physical activities.

Therapist (teacher) blinding: Subjects are assigned by an independent team (pedagogical authority); the therapist (teacher) is informed only of the standardized procedure and does not have access to sub-group information; a scripted intervention discourse is used, and suggestive language is avoided.

Data assessment blinding: Efficacy assessors (scale raters and fitness test raters) were exposed only to anonymized data to exclude the influence of subjective tendencies; data entry was double-checked to prevent leakage of time information.

Research matters process: The entire Project intervention was planned for 5 years, starting in September 2025 and ending in September 2030; the time phases were divided into the following: t_1_ September 2025, t_0_ October 2025, t_1_ February 2026, t_2_ June 2026, t_3_ February 2027, t_4_ June 2027, t_5_ September 2025. See Figure 2.

#### 2.3.3 Baseline characterization

Baseline measurements were completed prior to randomization into groups.

Indicators of the included baseline characteristics consisted of four components: (1) demographic indicators; (2) physical literacy indicators; (3) academic proficiency/physical fitness tests; and (4) post-intervention satisfaction. See Figure 2.

Demographic indicators, including general information about the participant, specifically school number, name, gender, age, grade level, major, alternative sport, email address, and phone number.

Academic level/physical fitness test, including physical form indicators, physical function indicators and physical fitness indicators, adopting the standards of China National Physical Fitness Monitoring University Student Group, in which the physical form indicators include height, weight and BMI; the physical function indicators include lung capacity; and the physical fitness indicators include seated forward bending, 50-m run, 800-m run (female)/1,000-m run (male), standing long jump, and pull-ups (male)/1-min sit-ups (female). Special Note: The inclusion of physical fitness test data as an indicator of academic level stems from regulations established by the Ministry of Education—the highest administrative authority in China's physical education system—which stipulate that passing a physical fitness test constitutes the basic standard for evaluating university students' academic level. Accordingly, this study incorporates physical fitness test data to represent university students' academic level in the Haian Flower Drum Project, thereby ensuring the research's universal value and replicability (13, 14).

Physical Literacy Indicators, incorporating the 18-item Perceived Physical Literacy Instrument (PPLI, 5-point Likert scale, Cronbach's alpha: 0.79–0.83). Based on the results of the Theory of Perceived Behavior and relevant previous studies (25), the PPLI will be divided into 2 sub-indicators, 6 sub-sub-indicators described as individual situational indicators (motivation, physical competence, and self-confidence) as well as collective situational indicators (interacting with the environment, expression, and understanding of knowledge). The eight sub-categories of the PPLI will be used to assess the sensitivity analyses for the structural model of the health behavior change.

The physical activity indicator, in the form of a questionnaire (yes or no), incorporates 4 sub-indicators, namely, time, speed, distance, and energy expended. The physical activity indicator was used as one of the baseline characteristics to assess data homogeneity. Specific Note: Physical activity metrics are not measured for Control group A.

#### 2.3.4 Outcome measurements

This study employs a two-arm design, with outcome measures for the experimental group and control groups A and B.

The outcome measures for all groups (t1–t4) were categorized in the same way as the baseline measures (t0) to explore the validity of the program intervention or structural model.

Physical activity data were collected using a gravimetric accelerometer (Pleasure Run App), as a tool, with sub-indicators calculated as the sum of values for each session. A special expression is needed for the speed sub-indicator, which is set to a negative value.

#### 2.3.5 Follow-up measurements

This study protocol was designed with five follow-up measurements after the start of the intervention to accomplish five secondary objectives. See Figure 2.

The first follow-up measurement, the baseline, is taken to accomplish Secondary objective 1.

A second follow-up measurement, taken at the post-distribution (t2) time point and measuring the same indicators as the outcome indicators, was used to fulfill Secondary objective 2.

The third and fourth follow-up measurements, with post-intervention satisfaction validation at the post-distribution (t3 and t4) time points, were conducted to complete Secondary objectives 3 and 4.

Post-intervention satisfaction was used to validate the extrinsic effects of health behavior change among university students at post-intervention, as well as to observe the psycho-structural characteristics of the health behavior change process among university students, including the wellbeing sub-indicator, the self-efficacy sub-indicator, the social support sub-indicator, the health perceptions sub-indicator, and the stress perceptions sub-indicator.

The wellbeing sub-indicator incorporated the Five-Item World Health Organization Wellbeing Index (6-point Likert scale: 0–5, Cronbach's alpha: 0.83–0.92) (29). Self-efficacy sub-indicator, incorporated the 8-item motivated strategies for learning questionnaire (MSLQ; 7-point Likert scale, Cronbach's alpha: 0.73–0.81) (30). Social support sub-indicator, incorporated into the 23-item social support questionnaire (5-point Likert scale, Cronbach 0.89) (31). Health perception sub-indicator incorporated a short 10-item generalized multicultural quality of life index (10-point Likert scale, Cronbach's alpha 0.73) (32). Stress perception sub-indicator, included in the Four Items Perceived Stress Scale (4-point Likert scale, Cronbach's alpha: 0.85) (33).

It needs to be clearly expressed that at both post-allocation (t3 and t4) time points, the academic level indicator and the physical activity indicator, although included in the measurements, are not included in the calculations, but are only used as observational indicators of the effectiveness of the intervention (judgment of status eligibility).

The fifth follow-up measurement was conducted in three phases between t4-t5 (3 years after the end of the intervention). This 3-year single-arm longitudinal cohort follow-up was conducted exclusively for the experimental group, with physical literacy data collected at 6th, 18th, and 36th months after the end of the intervention. The aim was to conduct an exploratory analysis of time-series psycho-structural characteristics in the health behavior changes of university students, thereby fulfilling Secondary objective 5.

#### 2.3.6 Standard operating procedures

The standard operating procedure for this study protocol consists of three parts: intervention operation, test diagnosis, etiologic study, and prognostic follow-up.

The first part is the standard protocol for treatment operations.

The first step is qualification and ethics, which mainly includes that teacher participants need to have a professional qualification from a college (sports) teacher. Before the experiment, it passed the ethics committee's review (including informed consent and risk planning).

The second step is the operation of the treatment phase: (1) the preparation period, the establishment of the therapeutic alliance, the first meeting to clarify the objectives, the principle of confidentiality and the responsibility of both parties; signing the informed consent (including the right to withdraw from the statement); (2) the core treatment period, the frequency of three times a week for 90 min each time, technical specifications need to be recorded in the health behavior change remodeling log; (3) the end of the period, to develop the prevention of recurrence and long-term plan.

The third step is the course setting: 64 weeks of long-term therapy, divided into four phases (each phase of 16 weeks in t0–t4 (between the first semester of the 2025–2026 academic year, and the second semester of the 2026–2027 academic year; total four semesters and 2 years).

The second part is a standardized protocol for diagnostic tests.

The first step was to standardize the instruments: Core scales included the Perceived Physical Literacy Instrument, the Happiness Questionnaire, the Self-Efficacy Questionnaire, the Social Support Questionnaire, the Health Perceptions Questionnaire, and the Stress Perception Questionnaire.

The second step is to prepare auxiliary tools: cell phone terminal app (EXCEL and Yue Running Circle) system, physical fitness test instrument (Zhongti Tongfang brand).

The third step is a specific procedure: it is divided into five sub-steps; first sub-step A (initial screening) –> B (baseline assessment); second sub-step B –> C (enrollment eligibility); third sub-step C –> |Yes | D (treatment implementation); fourth step C –> |No | E (referral or exclusion); and fifth sub-step D –> F (dynamic monitoring scale); baseline diagnosis needs to be completed in 1 week and symptom change was reassessed every 4 weeks in treatment.

The third part is a standard protocol for etiologic studies.

The first step was to determine the control variables: the independent variable was the academic level (data from the physical fitness test) of the Haian Flower Drum as an ICHSP, the dependent variable was the physical literacy, and the covariates were the TPPPAM (time, speed, distance, and energy expended by the physical activity).

The second step is the causal inference method: (1) Time series analysis: To verify the temporal association between the intervention and the change in symptoms, t0-t4, the independent variables, the strain variables, and the covariates. (2) Dose-effect test: Correlation (analytic factor) analysis of the intervention of treatment and efficacy, both feasibility and sensitivity analysis of HFDP based on SEVT.

The fourth part is the standard protocol for prognostic studies.

The first step was the follow-up design: (1) The time points were 16 weeks (t1)/32 weeks(t2)/48 weeks (t3)/64 weeks (t4) to 5 years (t5, at 6, 18, and 36 months after the end of the intervention) after the start of treatment. (2) Indicators were psychosocial indicators, and the included instruments were PPLI, wellbeing questionnaire, self-efficacy questionnaire, social support questionnaire, health perception questionnaire, and stress perception questionnaire.

The second step is bias control: (1) Compensation for lost visits: Benefiting from advances in information technology, this study utilized a web-based computing platform (the QQ app) to establish and maintain uniform management standards and improve retention rates. (2) Blinded assessment: Follow-up visits were performed by collectors of the experimental group who were not aware of the sub-groups.

### 2.4 Data collection, management, and analysis

#### 2.4.1 Analytical tools

The main data analysis tools included in this study protocol were Jamovi (version 2.6.26) as well as RStudio (version 4.5) (34).

#### 2.4.2 Sample size estimation

This research program recruited 608 university students for this study protocol. Based on an *a priori* efficacy analysis using Jamovi software, the number of participants required was determined by setting an F-test, ANOVA (repeated measures, within factors), δ0.25, power0.8, and α0.05 (34). This indicated that the study protocol would require a total sample size of N253. Based on N253, a 20% hypothesized dropout rate was considered. Therefore, an additional 13 participants were added to this estimated sample size (51 ≈ 253 × 20%). This finalized the total required sample size N608 [608 = (253 + 51) × 2]; the experimental group and the control group each comprised N304 participants; control groups A and B each comprised N152 participants. The dropout rate was chosen based on current physical activity criteria for university students.

This research design further examines the 10EPP criterion for sample size in structural model validation. The results for N608 satisfy this criterion: according to the 10EPP standard, a structural model with 5 parameters and an effect size of 0.2 requires a sample size of 500 [500 = (5 × 10) / 0.2 × 2; 500 < 608, satisfied].

This research protocol further examined the standard of having a sample size 5 to 20 times larger than the number of items in a questionnaire when conducting factor analysis or validity and reliability tests on the questionnaire alone. The results for N608 satisfied this requirement: among the six questionnaires, the Social Support Questionnaire had the majority of the items (*N* = 23); the experimental group had *N* = 304 (304 = 608/2), which is approximately 13.2 times the number of items (13.2 >5 and < 20, satisfied).

#### 2.4.3 Collection and management

The data collection and management of this research program consist of four components, namely, data collection specifications, source data management, personnel responsibilities, and the reconciliation system.

The first part, data collection specifications: first, to determine the collection content: (1) baseline data: demographic information, diagnostic scale scores, physiological indicators; (2) process data: treatment logs, behavioral observation records, intervention adherence reports; (3) outcome data: symptomatic improvement rate, functional assessment scales, adverse event records. Second, we determined the collection tools: online electronic common data system (EXCEL), real-time entry to avoid secondary transcription errors; paper-based forms need to be electronically completed within 24 h after double-checking.

The second part, source data management: first, determine the type of document: category examples of control measures; core documents; informed consent; the original scale stamped with a unique code; seal tamper-proof process document; experimental logs; encrypted storage; and access rights hierarchy. Second, to determine the preservation requirements: (1) Electronic data: Triple backup (local server + cloud + physical hard disk). (2) Paper documents: Special lockers, preservation period ≥5 years after the end of the study protocol.

The third part, personnel responsibilities: First, to determine the collection personnel: need to pass the GCP and psychology ethics training and assessment; prohibit single-person operation of key data (such as skill test records). Second, identify the entry personnel: independent of the treatment team, blinded entry; each batch of data should be labeled with the entrant's ID and time stamp.

The fourth part, the checking system: first, determine the three-level verification mechanism: graph LR, A [collector self-check] –> B [research assistant full check], B –> C [quality controller randomly sampling 10%]. Logically contradictory data need to be corrected retrospectively within 48 h. Next, determine the audit trail: all modifications record the original value, the modifier, and the reason.

#### 2.4.4 Statistical methods

In addition to the descriptive data analysis, this study protocol will analyze the changes in the primary outcome indicators from pre-intervention to post-intervention and at follow-up. To analyze the data for the primary outcomes, this study protocol will use linear mixed models (35). This type of modeling is particularly useful in settings where repeated measures (longitudinal studies) are made on the same participants. Linear mixed models also contain fixed and random effects. Since this study protocol expects a 20% dropout rate [using Intention-to-Treat (ITT) analyses, and comparing to Per-Protocol (PP) analyses], a particular advantage of linear mixed models is how they handle missing values. Participants with partial datasets could be included in the analysis. In testing hypotheses, the significance level used will be 5% (α = 0.05). Models will be adjusted for baseline scores on outcomes and covariates, including TPPPAM (time, speed, distance, and energy expended for physical activity).

A special expression is needed for the speed sub-indicator, which is set to a negative value.

To analyze the data for the secondary outcome, this study protocol incorporated hypothesis testing, specifically the inclusion of non-parametric variables (Spearman correlation), where a positive value indicates a change in the same direction of the variable (positive correlation), and a negative value indicates a change in the opposite direction (negative correlation), and *P* < 0.05 confirms the correlation. The detailed expression is the correlation between post-intervention satisfaction (wellbeing, self-efficacy, social support, health perceptions, and stress perceptions) and physical literacy, validating the feasibility and sensitivity of HFDP (36). This study protocol will also use validated factor analysis, combined with SEVT, to exploratively analyze the psycho-structural characteristics of the health behavior change process in college students, including the two dimensions of physical literacy (individual and environmental), 6 factors, and 18 entries (37).

In the structural equation model, this study treated social expectations as a method factor, using the brief Marlowe-Crowne Social Expectations Scale (MCSDS-13) as a common strategy to identify and correct common method variance resulting from social desirability bias (38). This approach allowed the present study to partially attribute measurement error to a latent method effect, thereby improving the accuracy of the estimated relationships between constructs in the structural model and mitigating the subjectivity inherent in questionnaire-based measures (the Perceived Physical Literacy Tool and satisfaction surveys).

### 2.5 Ethics and dissemination

The study protocol involving humans was approved by the Ethics Committee of Zhengye College (No. ZD-2025PE-003) and registered with the Chinese Clinical Trial Registry (Registration No. ChiCTR2500110409). Participants (university students from three universities) provided written informed consent. The study protocol was conducted in accordance with local legal and institutional requirements.

Privacy and data use/dissemination: Continuation of treatment data required reauthorization for inclusion in study protocol analyses; sample destruction or transfer required investigator consent.

The results of this research protocol will be progressively published publicly in academic journals or disseminated among stakeholders according to the time course of the research phase.

## 3 Discussion

### 3.1 Anticipated contributions

HFDP, based on SEVT, provides a model of the structure of health behavior change to serve as strong evidence for health behavior change in early adulthood.

The strain physical literacy theory, a popular topic in physical activity-thematic research, has not been found to have allosteric effects (2 stages, 6 dimensions, and 18 entries) during cross-cultural validation across all ages. The present study protocol will likely observe a physical literacy all-cause formation effect in health behavior change through a multi-subject intervention in physical activity in early adulthood. This may be a valid addition and refinement of physical literacy theory (literature).

A paradigm for more diverse physical education and teaching through a simple contextualized TPPPAM as a covariate of the health behavior change structural model. This study may serve as a model for policymakers in public health.

The data results provided in this study protocol on the sensitivity of health behavior change structural modeling will provide strong evidence of SEVT in early adult health behavior change (individual situational and collective situational). This may provide a baseline of practicality for subsequent multicenter studies of physical activity more broadly.

The Haian Flower Drum serves as a strong sub-intervention indicator for this study protocol, and the cultural attributes of the ICHSP in particular may be more palatable and acceptable to university students in early adulthood. This may provide a database for research on topics such as health behavior change in early adulthood, or even health-seeking behaviors, for a motivational type of thematic study.

Compared to general physical activities, as a form of intangible cultural heritage, Haian Flower Drum may, due to its high cultural vitality, exhibit the sensitivity described by the perception-behavior theory—where high perception leads to high physical activity—thereby facilitating healthy behavioral changes in early adulthood (39).

Compared to ethnic sports, as a form of intangible cultural heritage, Haian Flower Drum is more readily associated with cultural identity within its specific region, which, in turn, shapes a sense of identity. This provides solid evidence for the cultural-psychological dual construction of healthy behavioral changes in early adulthood (40).

### 3.2 Limitations

It is objectively recognized that this study protocol has some limitations. First, even with the rigorous scientific design of this study protocol, it was found that health behavior change may lead to two adverse events and two adverse reactions. Second, participants relied on gravity acceleration recorders to accomplish data recording during data collection in physical activity, which has some limitations (the timeliness of recording, which requires punctual attention from data collectors). Third, regarding methodological issues such as sample selection bias (which may give rise to the “Hawthorne effect,” where participants alter their behavior due to being observed) and the high dropout rate of 20% during the follow-up phase after the intervention ended (although mechanisms were in place to control for bias and compensate for nonresponse, and the social expectation method factor was tested), these still have certain limitations.

### 3.3 Advantages

First, cultural activities based in ICHSP trigger physical activity and promote health behavior change; this can stabilize motivation for health behaviors in early adulthood.

Second, TPPPAM, as an important factor in health behavior change, provided the audience (teacher participants and university student participants) with a clear visualization of physical activity/health behavior; this stabilized the audience's mood to participate in the experiment and, indirectly, solidified the feasibility and efficiency of the experiment.

## 4 Conclusion

As a pilot study, the SEVT-based HFDP will provide data on the feasibility and sensitivity of a structural model of health behavior change. It may also provide strong evidence of the psychological structure of health behavior change in early adulthood through data on efficacy.
